# A Mouse-Adapted CHIKV Strain Harboring E2-K200R and Non-Structural Mutations Exhibits Enhanced Pathogenicity in Multiple Rodent Models

**DOI:** 10.3390/v18040459

**Published:** 2026-04-12

**Authors:** Cong Tang, Bai Li, Qing Huang, Yun Yang, Wenhai Yu, Yanan Zhou, Daoju Wu, Hao Yang, Haixuan Wang, Junbin Wang, Shuaiyao Lu

**Affiliations:** 1 Institute of Medical Biology, Chinese Academy of Medical Sciences and Peking Union Medical College (IMBCAMS&PUMC), 935 Jiaoling Road, Kunming 650118, China; tangcong20210317@163.com (C.T.); libai119236714@126.com (B.L.); qinghuangfgh@163.com (Q.H.); yangyun.imbacms@outlook.com (Y.Y.); wenhaiyu1234@163.com (W.Y.); 15887034059@163.com (Y.Z.); wudaoju@imbacms.com.cn (D.W.); 15911606461@163.com (H.Y.); wanghaixuan@imbcams.com.cn (H.W.); 2 Yunnan Key Laboratory of Cross-Border Infectious Disease Control and Prevention and Novel Drug Development, Institute of Medical Biology, Chinese Academy of Medical Sciences & Peking Union Medical College, Kunming 650118, China; 3 State Key Laboratory of Respiratory Health and Multimorbidity, Chinese Academy of Medical Sciences & Peking Union Medical College, Beijing 100730, China; 4 Key Laboratory of Pathogen Infection Prevention and Control (Peking Union Medical College), Ministry of Education, Chinese Academy of Medical Sciences & Peking Union Medical College, Beijing 100730, China; 5 Yunnan Provincial Key Laboratory of Vector-borne Diseases Control and Research, Institute of Medical Biology, Chinese Academy of Medical Sciences & Peking Union Medical College, Kunming 650118, China

**Keywords:** Chikungunya virus, E2 protein, K200R mutation, adaptive evolution, animal models

## Abstract

Chikungunya virus (CHIKV) pathogenesis research has long been constrained by the lack of suitable immunocompetent rodent models. Through serial passaging in A129 and C57BL/6 mice, we obtained an adapted strain (CHIKV-Adapt) harboring an E2-K200R substitution along with non-structural protein mutations. Phenotypic analysis in C57BL/6 mice, BALB/c mice, and hamster models demonstrated that compared to the wild-type virus CHIKV-Adapt induced significantly higher and more prolonged viremia, broader tissue tropism, and more severe internal joint inflammation, without exacerbating external swelling. Notably, the K200R mutation did not alter the viral replication kinetics in vitro and was predicted not to affect its binding pattern to the MXRA8 receptor. Furthermore, mice challenged 160 days after primary infection exhibited nearly complete protective immunity. These findings indicate that E2-K200R is a critical adaptive mutation that, together with accompanying non-structural mutations, significantly enhances CHIKV replication capacity and pathogenicity in immunocompetent rodents without changing its in vitro replication ability or predicted receptor-binding mode. The acquisition of this adapted strain provides a new tool for CHIKV pathogenesis research and vaccine evaluation.

## 1. Introduction

The Chikungunya virus (CHIKV) is an enveloped RNA virus primarily transmitted by Aedes aegypti and Aedes albopictus mosquitoes, posing a persistent threat to global public health [1]. Infection typically leads to chikungunya fever, whose hallmark symptom is severe and persistent joint pain, often lasting for months or even years. Other common manifestations include fever, rash, headache, muscle pain, and fatigue. Although the disease is rarely fatal, it may cause serious complications, with higher risks particularly for the elderly, infants, and individuals with underlying health conditions [2]. Since the early 21st century, CHIKV has caused multiple large-scale outbreaks, and its geographic range has continually expanded, affecting over 110 countries and regions across the Americas, Africa, Asia, and Europe [3]. The Chikungunya virus results in approximately 16.9 million annual infections globally, with about 2.8 billion people living in high-risk transmission areas [4]. However, research on the pathogenesis of CHIKV has long been constrained by the lack of suitable animal models. Apart from newborn mice, wild-type adult mice are generally not susceptible to CHIKV infection. As a result, immunodeficient mouse strains (such as A129 and IFNAR^−^/^−^) have become the predominant models for research [5]. These models fail to recapitulate the full immune response of immunocompetent hosts. Therefore, generating a CHIKV strain that can efficiently replicate and cause disease in immunocompetent wild-type mice is imperative for studying its pathogenesis and host immunity under more physiologically relevant conditions.

The envelope of CHIKV is studded with heterodimeric spikes formed by E1 and E2 glycoproteins [6,7]. The E2 protein plays a central role in viral attachment to host cells, receptor recognition, and the induction of neutralizing antibodies [8,9,10,11]. Studies have shown that the E2 protein is a key determinant of viral host adaptation and pathogenicity [8]. A single amino acid mutation can significantly alter CHIKV’s cell tropism, transmission efficiency, and virulence in specific hosts. The well-known E1-A226V mutation directly contributed to the outbreak of CHIKV in the Indian Ocean region by enhancing viral replication and transmission efficiency in Aedes albopictus mosquitoes [12]. Similarly, other residues in the E2 protein, such as G55R, S119G, L182M, G206D, S300N and A345T, have also been reported to be closely associated with viral properties like neuroinvasiveness and resistance to neutralizing antibodies [13,14]. These findings highlight the critical importance of studying the functional consequences of natural variations in the E2 protein for understanding viral evolution and pathogenic mechanisms.

In a laboratory setting, simulating the natural evolution of viruses through serial in vivo passage is a classic strategy for identifying such key adaptive mutations [15]. This method applies selective pressure on the virus, enabling the screening of viral variants with a replication advantage in specific host environments, thereby providing valuable insights into the molecular basis of virus–host interactions [16].

In this study, through serial in vivo passages of CHIKV in A129 and C57BL/6 mice, we successfully isolated an adaptive strain carrying the E2-K200R mutation along with additional mutations in the non-structural proteins NSP1 (G230R) and NSP2 (M466E). We confirmed that these mutants exhibit significantly enhanced replication capacity and pathogenicity in various immunocompetent rodent models, including C57BL/6, BALB/c, as well as hamsters. In vitro cell models showed that the K200R mutation did not alter viral replication kinetics, and structural simulation analysis suggested that it did not significantly affect the predicted binding mode with the MXRA8 receptor. The acquisition of this adaptive strain and the preliminary exploration of its mechanisms provide a valuable tool and novel insights for further investigating CHIKV pathogenesis and host immune protective responses in immunocompetent animal models.

## 2. Materials and Methods

### 2.1. Animals and Ethics Statement

All animals used in this study were obtained from the Institute of Medical Biology, Chinese Academy of Medical Sciences, and Peking Union Medical College. (License No. SCXK (Dian) 2022-0002). Animal experiments were conducted in a Biosafety Level 3 laboratory under controlled conditions (temperature: 22–25 °C; relative humidity: 40–60%; 12 h light/dark cycle). All animal procedures were conducted in accordance with the guidelines of the Institutional Animal Care and Use Committee and approved by the Animal Ethics Committee of the Institute of Medical Biology, Chinese Academy of Medical Sciences (Approval No. DWSP2025O8001).

### 2.2. In Vivo Passaging and Adaptive Strain Isolation

The CHIKV wild-type (CHIKV-WT) strain (GenBank accession no. MH670649) was isolated in 2019 by the Yunnan Institute of Parasitic Diseases from an imported case in Ruili, China. The passaging procedure was as follows: 10^4^ plaque-forming units (PFU) of CHIKV-WT were used to infect A129 mice (6–8 weeks old, female) via hind foot ankle joint injection. Blood was collected at 2 days post-infection (dpi), and 50 μL of the serum was inoculated into C57BL/6 mice (6–8 weeks old, female) via hind foot ankle joint injection. This process was repeated for five passages in C57BL/6 mice, with 50 μL of serum collected at 2 dpi transferred to the next mouse at each passage. Finally, serum from the 5th passage was cultured in Vero cells for 2 rounds to amplify and stabilize the viral population. The resulting virus was designated CHIKV-Adapt. Whole-genome sequencing of the virus before and after passaging was performed using next-generation sequencing (DNBSEQ-T7). Sequence alignment and phylogenetic analysis were performed using MEGA 11, with multiple sequence alignment conducted using the Clustal W algorithm. Phylogenetic reconstruction was performed using the General Time Reversible model with a Gamma distribution and invariant sites to model rate variation. The robustness of the phylogenetic topology was evaluated by bootstrap analysis. The complete genome sequence of CHIKV-Adapt has been deposited in the GenBank database under the accession number PZ163663.

### 2.3. Structural Modeling and Molecular Docking

Based on the CHIKV E2-E1 structure (PDB ID: 6JO8) obtained from the PDB database, molecular docking was performed using AlphaFold3 to model the interactions between the E2 proteins of both CHIKV-WT and CHIKV-Adapt and the extracellular domains of mouse and human MXRA8, respectively. The resulting models were visualized and analyzed using PyMOL Molecular Graphics System (Version 2.5.0).

### 2.4. Analysis of Viral Replication Kinetics In Vitro (qPCR Method)

To evaluate viral replication ability in vitro, Vero cells (from our laboratory stocks) and A549 cells (from our laboratory stocks) were seeded in 96-well plates at a density of 2 × 10^4^ cells per well. Vero cells were cultured in DMEM containing 10% fetal bovine serum (FBS), and A549 cells were cultured in F-12K basal medium + 10% FBS. The cells were cultured overnight. The next day, cells were infected with CHIKV-Adapt or CHIKV-WT at an MOI of 0.05 and incubated at 37 °C for 1 h for viral adsorption. The inoculum was then removed, cells were washed twice with PBS, and maintenance medium (DMEM with 2% FBS) was added. Cell culture supernatants were collected at 24-, 48-, and 72-h post-infection. Viral RNA was extracted from the supernatants using TRIzol reagent, and one-step real-time quantitative RT-PCR (qRT-PCR) was performed to detect viral RNA copy numbers according to the method described in ‘Materials and Methods Section 2.6’. Three biological replicates were set for each time point, and the experiment was independently repeated twice. Viral replication levels were expressed as the logarithm of viral RNA copies per milliliter of supernatant (Log_10_ copies/mL), and growth curves were plotted.

### 2.5. Animal Infection Experiments

The susceptibility to infection was evaluated using C57BL/6 mice, BALB/c mice, and hamsters (all aged 6–8 weeks, female). Animals were inoculated subcutaneously in the hind limb with 10^5^ PFU of either CHIKV-Adapt or CHIKV-WT. Blood and tissue samples (including muscle, joint, and spleen) were collected at 5 dpi for viral load quantification and histopathological analysis (H&E staining). The degree of ankle swelling was measured daily using a caliper. BALB/c mice that had undergone primary infection with CHIKV-Adapt were rechallenged with an identical dose (10^5^ PFU) of CHIKV-Adapt 160 days later, and both blood and tissue viral loads were measured following the challenge.

### 2.6. Quantitative PCR (qPCR)

Tissue samples were homogenized in 800 μL of TRIzol reagent, and 200 μL of the supernatant was used for RNA extraction. For whole blood samples, 50 μL of blood was mixed with 200 μL of TRIzol by vortexing, and the mixture was used for RNA extraction. For cell culture supernatant samples, 200 μL of supernatant was mixed with 600 μL of TRIzol by vortexing, and the mixture was used for RNA extraction. RNA was extracted using the Zymo zol™ RNA MiniPrep Plus kit (R2052) according to the manufacturer’s instructions. Viral load was quantified using the TaqMan Fast Virus 1-Step Master Mix on a Bio-Rad CFX384 Touch system. Viral loads are expressed as viral copies per gram of tissue per milliliter of blood, or per milliliter of cell culture supernatant. Following gradient dilution plasmids containing the target were used as standard samples. One-step RT-qPCR protocol was as follows: 25 °C for 2 min, 50 °C for 15 min, and 95 °C for 2 min; followed by 40 cycles of 95 °C for 5 s and 60 °C for 31 s. The primer and probe sequences for detecting CHIKV were: CHIKV-F: 5′-AAGCTCCGCGTCCTTTACCAAG-3′, CHIKV-R: 5′-CCAAATTGTCCTGGTCTTCCT-3′, CHIKV-Probe: 5′-FAM-CCAATGTCTTCAGCCTGGACACCTTT-TAMRA-3′.

### 2.7. Histopathological Analysis

Tissue samples were fixed in 10% neutral buffered formalin, embedded in paraffin, and sectioned into 5-μm-thick slices for hematoxylin and eosin (H&E) staining. The stained sections were digitized using a 3DHISTECH panoramic slide scanner and evaluated by professional pathologists using CaseViewer software (v2.4). A semi-quantitative histopathological scoring system (0–3 for each parameter) was used to assess the severity of CHIKV-induced arthritis in murine joints. This scoring system, adapted from established comprehensive grading schemes for murine joint pathology [17], evaluated five parameters: synovial hyperplasia, inflammatory cell infiltration, joint effusion, soft tissue lesions, and cartilage surface alterations. The specific criteria for each grade are detailed in Supplemental Table S1. The total pathology score was calculated as the sum of the scores for these five parameters, resulting in a total score ranging from 0 to 15.

### 2.8. Neutralization Test (CPE Method)

The serum neutralizing antibody titers were determined using a cytopathic effect (CPE) assay. Mouse serum samples were first inactivated at 56 °C for 30 min, then serially diluted two-fold with DMEM in 96-well plates, with dilutions ranging from 1:16 to 1:2048. Each well contained 50 μL of the diluted serum. The virus was diluted with DMEM to 2000 PFU/mL, and 50 μL of the virus dilution was added to each well. After mixing with the serum, the plates were incubated at 37 °C for 1 h. Subsequently, 100 μL of Vero cell suspension (containing 1.5 × 10^4^ cells) was added to each well. After incubation for 5–7 days, cytopathic effect (CPE) was observed under a microscope to evaluate the neutralizing capacity of the sera. Neutralizing antibody titers were calculated using the Karber method, expressed as the reciprocal of the highest serum dilution that inhibited 50% of viral infection. Each dilution was tested in duplicate. If only one of the two wells showed CPE at a given dilution, the reciprocal of that dilution was recorded as the titer. If no CPE was observed in both wells at a dilution, the average of the reciprocals of that dilution and the next dilution was taken as the titer.

### 2.9. Statistical Analysis

Statistical analysis was performed using GraphPad Prism software (version 9.4.0). Data are presented as mean ± SEM, and comparisons between two groups were conducted using Student’s *t*-test. A *p*-value < 0.05 was considered statistically significant. Detailed descriptions are provided in the figure legends.

## 3. Results

### 3.1. CHIKV-Adapt Strain Obtained via In Vivo Passaging and Validation of Its In Vitro Replication Kinetics

**Figure 1:**
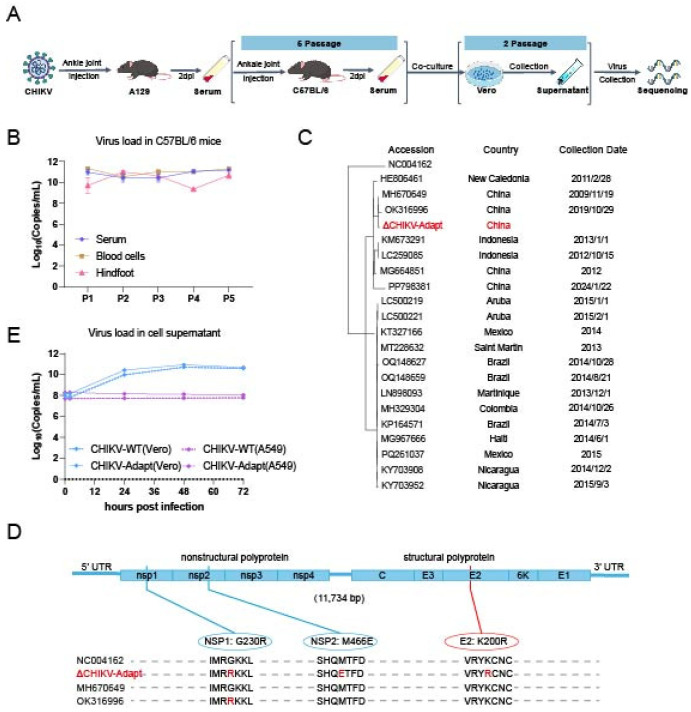
Generation, characterization, and in vitro replication kinetics of the CHIKV-Adapt strain.: (**A**) Schematic of the generation procedure for the CHIKV-Adapt strain; (**B**) Successful viral adaptation during passaging in C57BL/6 mice; (**C**) Phylogenetic analysis of CHIKV-Adapt (GenBank accession no. PZ163663) and the wild-type parental strain CHIKV-WT (GenBank accession no. MH670649) along with reference strains; (**D**) Genome structure of CHIKV-Adapt showing the locations of acquired mutations (E2-K200R, NSP1-G230R, NSP2-M466E); (**E**) Comparison of single-step growth curves of CHIKV-Adapt and CHIKV-WT in Vero and A549 cells. Data are mean ± SEM.

To obtain a CHIKV strain capable of infecting wild-type mice, we first passaged CHIKV-WT once in A129 mice. Subsequently, serum from the infected mice was used to perform five consecutive passages in C57BL/6 mice. The virus from the 5th passage was then cultured in Vero cells for two rounds to amplify and stabilize the viral stock, yielding the adapted strain named CHIKV-Adapt (Figure 1A). Successful viral adaptation was confirmed by detecting viremia at 2 dpi after each passage (Figure 1B). Whole-genome sequencing revealed that compared to CHIKV-WT (MH670649), CHIKV-Adapt (PZ163663) harbored three consistent mutations: a lysine-to-arginine substitution at position 200 of the E2 protein (E2-K200R), a glycine-to-arginine substitution at position 230 of NSP1 (NSP1-G230R), and a methionine-to-glutamic acid substitution at position 466 of NSP2 (NSP2-M466E). Phylogenetic analysis confirmed that these mutations were specific to this passaging process (Figure 1C,D). To assess the impact of the K200R mutation on basic replicative capacity, we compared the growth kinetics of CHIKV-Adapt and CHIKV-WT in Vero and A549 cells. The results showed no significant difference in viral titers in the cell culture supernatant at the indicated time points post-infection (12, 24, 48, and 72 h) (Figure 1E), indicating that the K200R mutation does not directly enhance in vitro replication efficiency in the tested cell lines.

### 3.2. The E2-K200R Mutation Does Not Induce Significant Overall Structural Changes in the Envelope Protein

**Figure 2:**
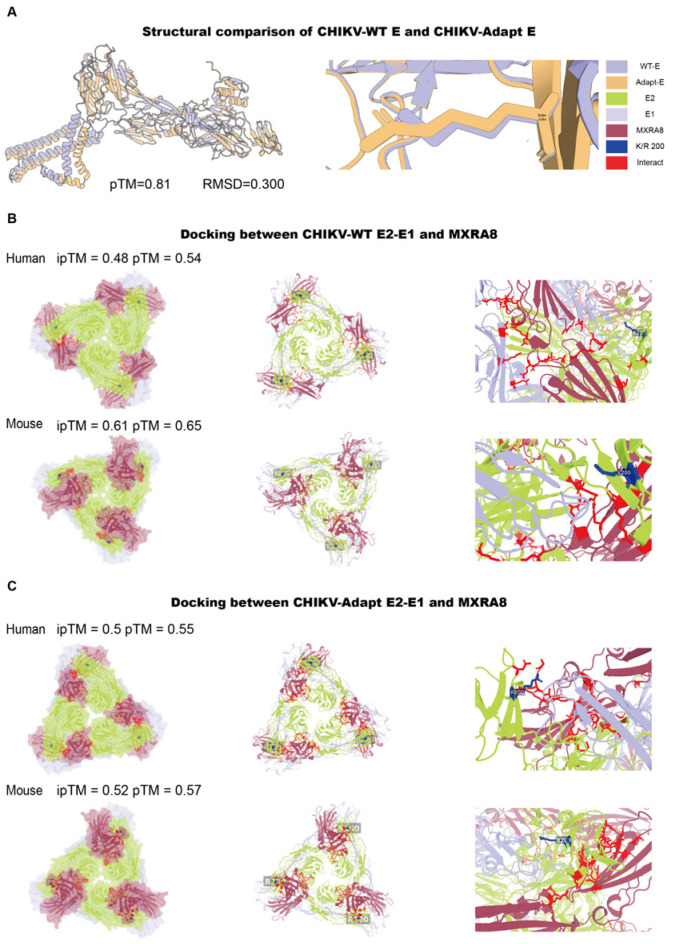
Structural and receptor-docking analysis of the CHIKV E protein. (**A**) Structural comparison of the mature E2-E1 heterodimer between CHIKV-WT and CHIKV-Adapt. (**B**,**C**) Molecular docking models of the E2 protein with MXRA8 receptors. (**B**) Docking between CHIKV-WT-E2 and human/mouse MXRA8. (**C**) Docking between CHIKV-Adapt-E2 and human/mouse MXRA8. The results indicate that the K200R mutation does not significantly alter the overall E protein structure or its predicted interaction with the MXRA8 receptor.

To investigate the impact of the K200R mutation on viral protein structure and function, we first performed three-dimensional structural modeling and comparison of mature E2-E1 heterodimer (based on the PDB entry 6JO8) from both viral strains. The results showed that both predicted models exhibited high confidence (pTM = 0.81), and their overall structural backbones were highly consistent (RMSD = 0.300 Å), indicating that the mutation did not cause significant conformational changes in the E protein (Figure 2A). On this basis, to determine whether the mutation affects virus-receptor interactions, we used AlphaFold3 to perform molecular docking simulations between the trimeric E2/E1 heterodimer of CHIKV-WT and CHIKV-Adapt and the ectodomains of human and mouse MXRA8. Analysis revealed that the trimeric E2/E1 heterodimers from both viruses formed stable complex models with MXRA8 from both species. All docking models showed favorable overall confidence scores (pTM: CHIKV-WT/hMXRA8: 0.54, CHIKV-Adapt/hMXRA8: 0.55; CHIKV-WT/mMXRA8: 0.65, CHIKV-Adapt/mMXRA8: 0.57) and interface prediction accuracy (ipTM: CHIKV-WT/hMXRA8: 0.48, CHIKV-Adapt/hMXRA8: 0.5; CHIKV-WT/mMXRA8: 0.61, CHIKV-Adapt/mMXRA8: 0.57), with no significant differences between the combinations (Figure 2B,C).

To further explore the potential impact of the K200R mutation on MARCO recognition, we similarly performed structural prediction of the E2-MARCO interaction using AlphaFold3. The prediction showed that the mutation did not induce significant conformational changes in the E2-MARCO binding interface, with comparable confidence scores between the two strains (Supplementary Figure S1). Collectively, these computational predictions suggest that the K200R mutation does not induce significant structural alterations in the E2-E1 heterodimer, nor does it affect the predicted binding mode or affinity for either the MXRA8 receptor or the MARCO scavenger receptor.

### 3.3. The E2-K200R Mutation Significantly Enhances Viral Susceptibility in Multiple Wild-Type Rodent Models

**Figure 3:**
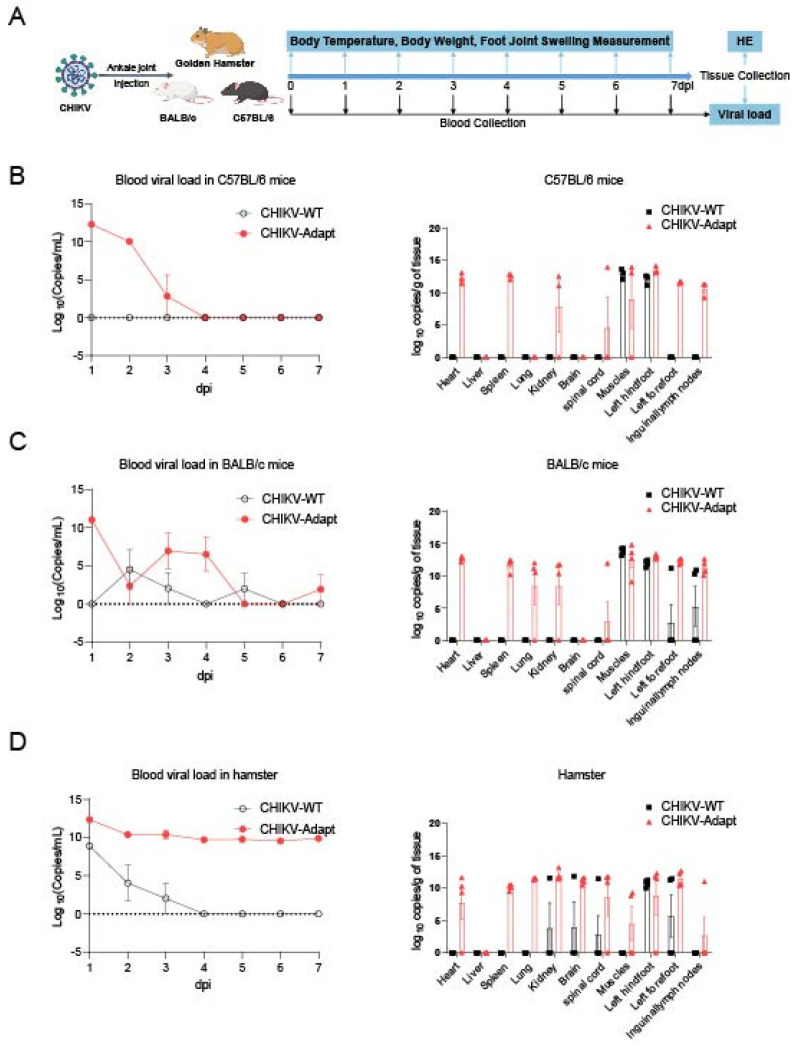
Enhanced pathogenicity of CHIKV-Adapt in three immunocompetent rodent models. (**A**) Schematic of the comparative infection experiment in C57BL/6 mice, BALB/c mice, and hamsters. (**B**–**D**). Comparative viral loads in blood and tissues of (**B**) C57BL/6 mice, (**C**) BALB/c mice, and (**D**) hamsters infected with CHIKV-WT or CHIKV-Adapt.

To comprehensively evaluate the pathogenicity of CHIKV-Adapt in vivo, we conducted parallel comparative studies in three immunocompetent rodent models: C57BL/6 mice, BALB/c mice, and hamsters. The results showed that compared to CHIKV-WT, CHIKV-Adapt induced stronger and more prolonged viremia in all models: in C57BL/6 mice, CHIKV-WT did not induce detectable viremia, while CHIKV-Adapt triggered evident viremia from 1 to 3 dpi (Figure 3A); in BALB/c mice, CHIKV-WT induced only weak and intermittent viremia (at 2, 3, 5 dpi), whereas CHIKV-Adapt caused higher peak viremia at 1, 3, and 4 dpi (Figure 3B); in hamsters, viremia was detectable for CHIKV-WT from 1 to 3 dpi, but CHIKV-Adapt not only achieved significantly higher viral loads but also maintained viremia until 7 dpi without clearance (Figure 3C). Beyond the enhanced systemic viremia, analysis of tissue viral loads further showed that CHIKV-Adapt exhibited broader tissue tropism and replication capacity: in C57BL/6 mice, CHIKV-WT infection was confined to muscle and the left hind foot, while CHIKV-Adapt additionally spread to the spleen, heart, kidney, spinal cord, left forefoot, and inguinal lymph nodes (Figure 3A); in BALB/c mice, CHIKV-WT distribution was limited to muscle, left hind foot, left forefoot, and inguinal lymph nodes, whereas CHIKV-Adapt further invaded the spleen, lung, heart, kidney, and spinal cord (Figure 3B); in hamsters, CHIKV-WT was detected only at low levels in the kidney, brain, spleen, and left hind foot, while CHIKV-Adapt reached higher viral loads in a wide range of tissues including the brain, muscle, lung, heart, spinal cord, and multiple lymph nodes (Figure 3C). Collectively, these results indicate that the K200R mutation not only significantly enhances viral replication and persistence in the circulatory system but also promotes viral dissemination to peripheral tissues and enables more efficient local amplification.

### 3.4. CHIKV-Adapt Enhances Joint Inflammation and Tissue Damage Without Exacerbating Clinical Swelling

**Figure 4:**
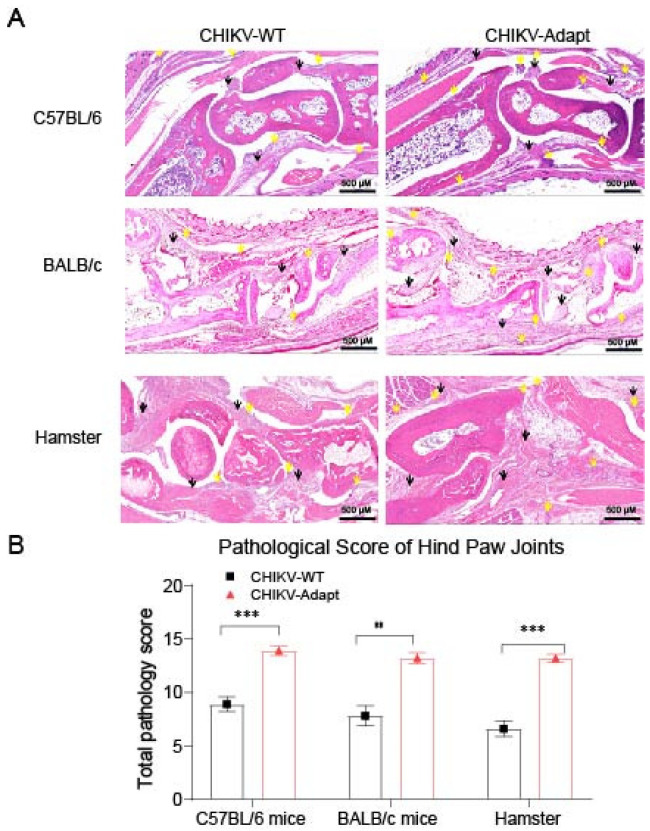
The K200R mutation exacerbates internal joint pathology. (**A**) Representative hematoxylin and eosin (H&E)-stained tissue sections of hind paw joints from C57BL/6 mice, BALB/c mice, and hamsters at 7 dpi with either CHIKV-WT or CHIKV-Adapt virus. Images show that CHIKV-Adapt infection induced more severe microscopic lesions, such as synovial hyperplasia and inflammatory cell infiltration, compared to CHIKV-WT infection across all animal models. Black arrowheads indicate synovial hyperplasia, and yellow arrowheads indicate areas of inflammatory cell infiltration. Scale bar: 500 µm. (**B**) The total pathology score represents the sum of scores for five parameters, each scored on a scale of 0–3 (total score range: 0–15). Data are presented as mean ± SEM. ** *p* < 0.01, *** *p* < 0.001.

To evaluate the pathogenic phenotype of the K200R mutation in vivo, we concurrently analyzed external joint swelling and internal pathology in C57BL/6 mice, BALB/c mice, and hamster models. Serial measurements of ankle joint diameters from 0 to 7 dpi revealed that CHIKV-Adapt did not induce more significant external joint swelling compared to CHIKV-WT (Supplementary Figures S2 and S3). However, histopathological assessment revealed severe internal damage. Notably, this damage was characterized by pronounced infiltration of mononuclear cells and lymphocytes into the joint tissues, along with synovial hyperplasia. As shown in Supplementary Table S2, across all three models, the mutant strain group showed significantly higher scores for these pathological parameters—specifically mononuclear/lymphocytic cell infiltration and synovial hyperplasia—than the wild-type group (all *p* < 0.05). These aggravated microscopic lesions are visible in tissue sections (Figure 4A) and are summarized as significantly higher total pathology scores (Figure 4B, *p* < 0.01 or <0.001). In summary, the K200R mutation can induce more severe internal joint inflammation and tissue destruction, but this enhanced pathogenicity does not translate into significant external swelling.

### 3.5. Primary Infection Induces Long-Lasting Protective Immunity

**Figure 5:**
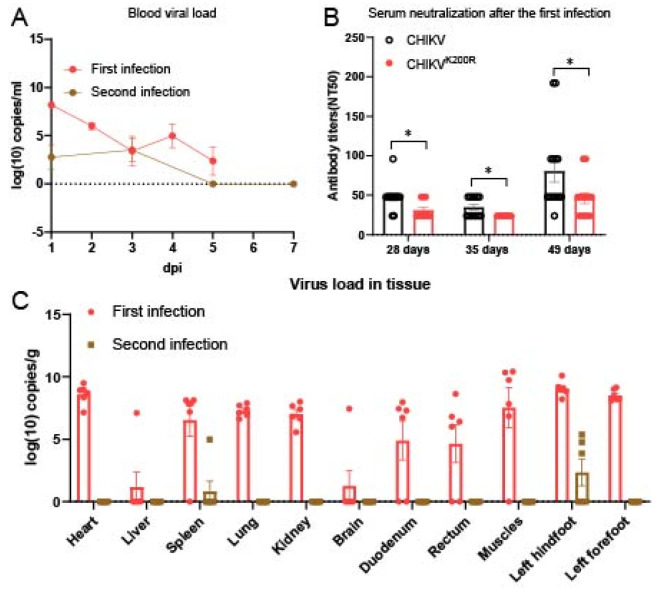
Primary CHIKV infection induces long-lasting protective immunity against homologous rechallenge. (**A**) Viremia post rechallenges. BALB/c mice were rechallenged with the homologous virus (CHIKV-Adapt) 160 days after primary infection. Viral RNA loads in whole blood were measured and compared to those during primary infection. Data are presented as mean ± SEM. (**B**) Serum neutralizing antibody titers were determined at 28, 35, and 49 days after primary infection using homologous virus combinations: sera from CHIKV-WT-infected mice were tested against CHIKV-WT, and sera from CHIKV-Adapt-infected mice were tested against CHIKV-Adapt. Data are presented as mean ± SEM. Statistical significance between groups at each time point was determined by Student’s *t*-test (* *p* < 0.05). (**C**) Tissue viral loads post rechallenges. Viral RNA loads in the indicated tissues (spleen, left hind footpad, and other major organs) were quantified following homologous rechallenge. Data are presented as mean ± SEM for tissues in which virus was detected.

To investigate the protective immunity induced by CHIKV-Adapt infection, we rechallenged BALB/c mice with the homologous virus 160 days after the primary infection. The results showed that compared with the primary infection, viremia in rechallenged mice was extremely low and nearly undetectable (Figure 5A). We also measured the neutralizing antibody levels in sera collected at 28, 35, and 49 days after primary infection with either CHIKV-WT or CHIKV-Adapt. The CHIKV-WT-infected group showed significantly higher neutralizing antibody titers than the CHIKV-Adapt group at these time points (*p* < 0.05) (Figure 5B). Regarding viral tissue distribution, apart from very low viral loads detected in the spleen and left hind footpad, no virus was detectable in other tissues (Figure 5C). These data indicate that primary infection induces strong and long-lasting immune memory capable of effectively controlling reinfection.

## 4. Discussion

Through independent in vivo passaging experiments, this study obtained an adapted strain harboring the E2-K200R mutation along with mutations in the non-structural proteins NSP1 and NSP2, and confirmed that these strains exhibit significantly enhanced in vivo replication capacity and pathogenicity in multiple immunocompetent animal models. Although E2-K200R is likely a key adaptive mutation, we recognize that the additional non-structural protein mutations may also contribute to shaping this phenotype. We found in multiple immunocompetent animal models, including C57BL/6 mice, BALB/c mice, and hamsters, that this mutation significantly enhanced viral replication in vivo without altering its in vitro replication kinetics or the predicted structure of the E2-E1 heterodimer. It also induced significant infiltration of mononuclear/lymphocytic inflammatory cells in the joints but did not cause noticeable external joint swelling, while simultaneously eliciting durable immune protection lasting up to 160 days.

The E2 protein-encoding gene is a hotspot for host-adaptive evolution, where a single amino acid substitution can alter viral properties [18,19,20]. The E2-K200R mutation identified in this study further supports this view. Notably, this mutation is not the first to be reported. Previous research has shown that CHIKV can also evolve variants carrying the E2-K200R or mutations at adjacent sites (e.g., E2-K200Q) during chronic infection in IFNAR^−^/^−^ mice. These mutations have been identified as key drivers enhancing the virus’s transmission capacity and pathogenicity in wild-type mice [21]. Our study, utilizing an independent experimental system involving serial passage from A129 to C57BL/6 mice, has reproduced and extended these finding. We systematically validated the phenotype of enhanced viremia and tissue viral loads across three immunocompetent models: C57BL/6 mice, BALB/c mice, and hamsters. This observed convergent evolution at the E2-K200 site under different laboratory conditions and selection pressures strongly supports its central role in CHIKV’s adaptation to the murine host. Unlike the well-characterized E1-A226V mutation, which primarily enhances mosquito transmission efficiency [12], the E2-K200R mutation appears to be more focused on overcoming mammalian host restrictions in vivo, reflecting the virus’s divergent evolutionary strategies when facing distinct host barriers.

Both the present study and prior work have found that the E2-K200R mutation significantly elevates viremia in vivo without affecting in vitro replication. This ‘in vivo specificity’ suggests an advantage stemming from interaction with the host’s internal environment. Based on previous studies, a plausible mechanistic explanation involves the scavenger receptor MARCO. Carpentier et al. demonstrated that wild-type CHIKV particles are recognized and rapidly cleared from the circulation by liver Kupffer cells via MARCO, which specifically interacts with the lysine (K) at position 200 of the E2 protein [21,22]. The K200R substitution allows CHIKV-Adapt to evade this innate immune clearance pathway, thereby providing a potential explanation for the prolonged viremia and enhanced systemic dissemination we observed across all rodent models. Interestingly, La Fleur et al. demonstrated that A549 lung cancer cells are involved in MARCO regulation, producing IL37 that induces MARCO expression on macrophages [23]. This indicates that A549 cells possess the molecular machinery to interact with the MARCO pathway. In light of this, the comparable replication kinetics of CHIKV-WT and CHIKV-Adapt in A549 cells suggest that the replication advantage conferred by the K200R mutation may not be simply cell-autonomous, but rather may depend on the complex in vivo microenvironment, particularly the liver microenvironment where MARCO-expressing Kupffer cells could play a key role in this mechanism. This interpretation aligns with the model that the mutation enhances virulence by evading MARCO-mediated clearance and underscores the value of our immunocompetent animal models for studying this mechanism. Our structural prediction that the K200R mutation does not significantly alter the E2-MARCO binding interface is consistent with the known limitation that AlphaFold3 may not accurately capture the functional impact of point mutations. This negative result does not contradict the well-established in vivo evidence for MARCO-mediated clearance [22]; rather, it highlights the need for experimental approaches such as surface plasmon resonance to definitively elucidate the molecular basis of MARCO evasion. Nevertheless, direct validation using primary macrophages or Kupffer cells remains the ideal approach and an important direction for future studies. Currently, there is no direct evidence indicating that this mutation significantly alters the binding pattern of the virus to its primary receptor, MXRA8. In recent years, the underlying molecular mechanism has been partially elucidated. Carpentier et al. discovered that wild-type CHIKV particles can be recognized and rapidly cleared by the scavenger receptor MARCO on the surface of liver Kupffer cells via the lysine (K) at position 200 of the E2 protein. In contrast, the E2-K200R mutation enables the virus to evade this clearance pathway, allowing viral particles to persist longer in the bloodstream, thereby establishing higher levels of viremia and accelerating systemic dissemination [22].

It is important to acknowledge that, in addition to the E2-K200R mutation, our adapted virus also carries mutations in the non-structural proteins NSP1 (G230R) and NSP2 (M466E). Alphavirus non-structural proteins play critical roles in viral replication and host immune evasion [24]. NSP1 is involved in RNA capping and replication complex formation, and its mutation could potentially affect the anchoring efficiency of replication complexes on intracellular membranes or the stability of viral RNA [24]. NSP2 functions as a helicase, protease, and antagonist of host innate immune responses [25], particularly through inhibition of interferon-induced JAK-STAT signaling [26] and induction of host transcriptional shutoff [27]. Therefore, the NSP2-M466E mutation might also influence viral pathogenicity or immune evasion in vivo. We recognize that the observed enhanced phenotype could result from the combined action of these three mutations rather than from E2-K200R alone. Ideally, generating recombinant viruses carrying individual mutations and their combinations through reverse genetics would be the gold standard for dissecting the relative contributions of each mutation. We plan to conduct such experiments in future studies to elucidate the functional roles of NSP1-G230R and NSP2-M466E relative to E2-K200R. Nevertheless, the convergent evolution at the E2-K200 site observed across independent studies [21] strongly supports its central role in CHIKV adaptation to murine hosts.

This study found that infection with the CHIKV-Adapt strain carrying the K200R mutation induced severe inflammatory infiltration dominated by mononuclear cells and lymphocytes in the joints, along with higher histopathological scores, but did not cause significant visible joint swelling. This phenotype of “dissociation between pathological damage and clinical swelling” provides a new perspective for understanding the pathogenesis of Chikungunya virus arthritis (CHIKF). In traditional models of arthritis, acute swelling is often directly associated with neutrophil infiltration and vasogenic edema [28]. The phenotype observed in this study suggests that the K200R mutation may drive a distinct inflammatory response pattern. This model simulates the clinical phenomenon observed in some CHIKF patients who experience severe joint pain and dysfunction without obvious visible swelling [29]. Based on existing literature, several hypotheses may explain this unique phenotypic dissociation. It could stem from differences in inflammatory cell recruitment: the mutation might preferentially recruit monocytes/lymphocytes via pathways such as CCL2/MCP-1, leading to tissue damage, rather than triggering massive neutrophil infiltration and acute edema through pathways like CXCL1/KC [30]. Of particular note, recent research has revealed a pathogenic interaction between macrophages and CD4^+^ T cells in CHIKV joint pathology, providing a potential specific immunological mechanism for explaining this mononuclear/lymphocyte-dominant inflammatory pattern [31]. These literature-based mechanistic hypotheses offer potential explanations for our observations but require direct experimental validation, including measurement of cytokines and immune cell dynamics, in future studies.

Parallel to the distinct pathological phenotype, we also observed a significant difference in humoral immune responses induced by the two viruses: when tested with homologous virus, neutralizing antibody titers in the CHIKV-WT-infected group were significantly higher than those in the CHIKV-Adapt-infected group at each time point. The distinct in vivo replication kinetics of the two viruses may explain this result: CHIKV-WT, despite poor replication in immunocompetent mice, may present antigens in a context favoring robust homologous neutralizing antibody responses, while the enhanced replication of CHIKV-Adapt might trigger immunoregulatory mechanisms that limit antibody production. Alternatively, the K200R mutation may alter E2 antigenicity, potentially reducing the immunogenicity of certain neutralizing epitopes. Further epitope mapping studies are needed to elucidate this phenomenon.

This study has several limitations. First, the specific molecular mechanisms by which the E2-K200R, NSP1, and NSP2 mutations affect virus–host interactions have not been fully elucidated. The enhanced in vivo replication capacity and unique pathological phenotype exhibited by these mutations suggest that they may function through complex pathways, such as influencing interactions with various host factors and regulating immune responses. These aspects require further exploration and validation in subsequent studies. Second, our molecular modeling analysis of the E2-K200R mutation’s effect on MXRA8 binding is based on theoretical predictions, and its biological significance still needs further experimental support. In addition, viral loads in this study were assessed by RT-qPCR, which sensitively quantifies viral RNA but cannot directly distinguish infectious viral particles. A more comprehensive assessment of viral replication capacity awaits the combined application of multiple detection methods. Addressing these questions will further advance our understanding of CHIKV host adaptation and pathogenesis.

## 5. Conclusions

This study not only independently validated the importance of E2-K200R as a key host-adaptive mutation for CHIKV but also yielded a CHIKV strain highly adapted to immunocompetent animal models, carrying additional mutations in NSP1 and NSP2. Its core phenotypic characteristics are enhanced in vivo fitness and a dissociation between significant inflammatory pathological damage in the joints and the absence of observable external swelling. E2-K200R, as one of the key sites, plays an important role in the viral adaptation process. This tool model will significantly advance research into the pathogenesis of CHIKV, particularly its chronic arthritis and sequelae. Our findings underscore the central, hub-like role of the E2 protein B-domain in CHIKV evolution and pathogenicity, providing novel scientific rationale and experimental tools for the future development of broad-spectrum vaccines and antiviral strategies targeting this critical region.

## Data Availability

The original contributions presented in this study are included in the article/Supplementary Materials. The genome sequence of the adapted CHIKV strain (CHIKV-Adapt) generated in this study has been deposited in GenBank under accession number PZ163663 and will be made publicly available upon publication.

## Supplementary Materials

The following supporting information can be downloaded at: https://www.mdpi.com/article/10.3390/v18040459/s1, Supplemental Table S1 (Semi-quantitative histopathological scoring criteria for murine joint pathology upon CHIKV infection), Supplemental Table S2 (Summary of semi-quantitative histopathological scores across different rodent models), Supplemental Figure S1 (Structural simulation of the interaction between the E2-E1 heterodimer and MARCO), Supplemental Figure S2 (Rearfoot ankle joint swelling in CHIKV rodent models), Supplemental Figure S3 (Forefoot ankle joint swelling in CHIKV rodent models).

## Abbreviations

The following abbreviations are used in this manuscript:

CHIKV	Chikungunya virus
CHIKV-Adapt	Chikungunya virus adapted strain
CHIKV-WT	Chikungunya virus wild-tape strain
CHIKF	Linear dichroism Chikungunya virus arthritis
