# Supplementary material for: A Mouse-Adapted CHIKV Strain Harboring E2-K200R and Non-Structural Mutations Exhibits Enhanced Pathogenicity in Multiple Rodent Models
Source: Viruses. 2026 Apr 12;18(4):459. doi: 10.3390/v18040459 (PMC13120490; doi:10.3390/v18040459)
Supplement: Supplementary file 1 [file viruses-18-00459-s001.zip › Supplementary Figures S1-S3.pdf]

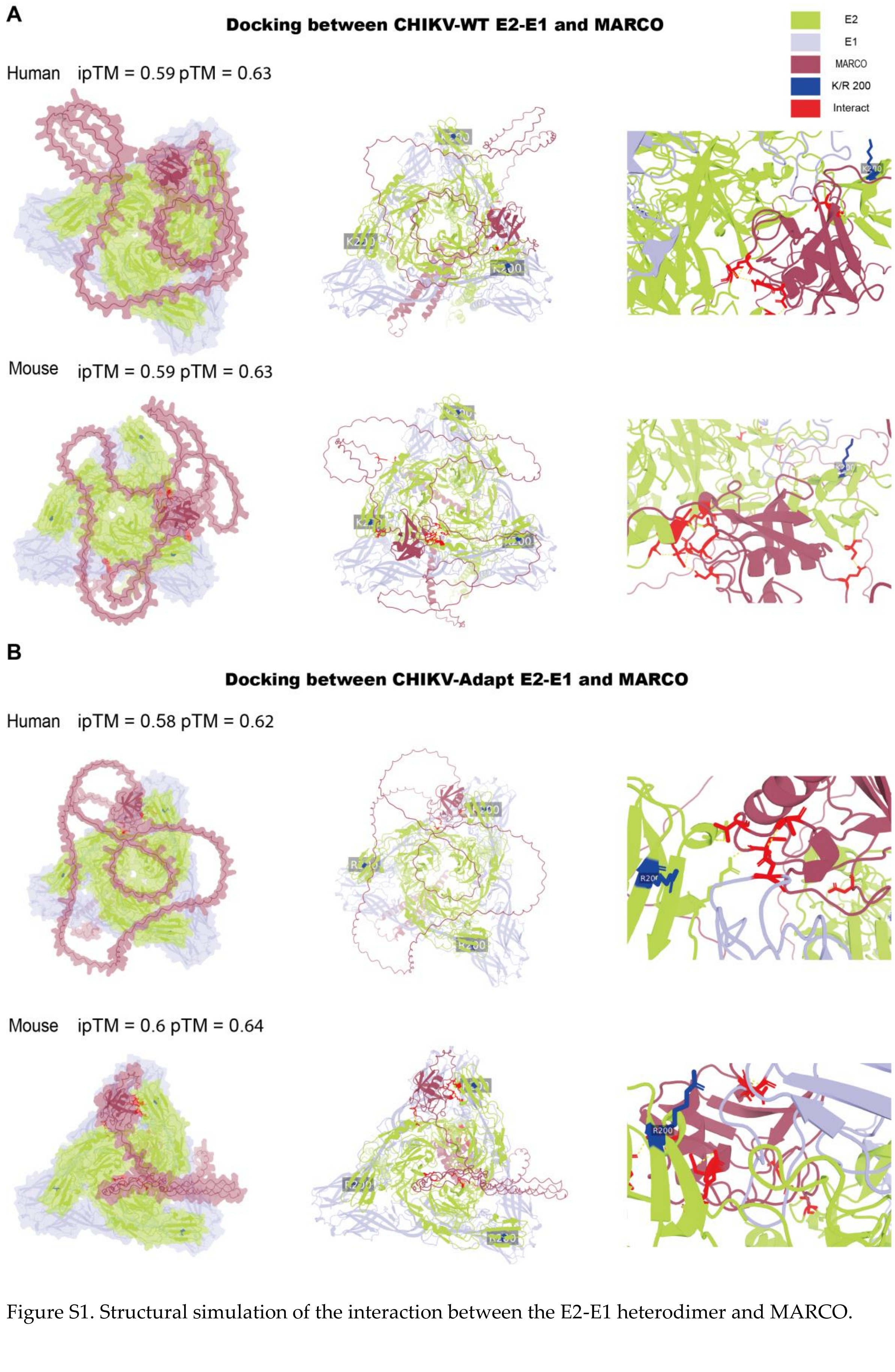


Figure S1. Structural simulation of the interaction between the E2-E1 heterodimer and MARCO.

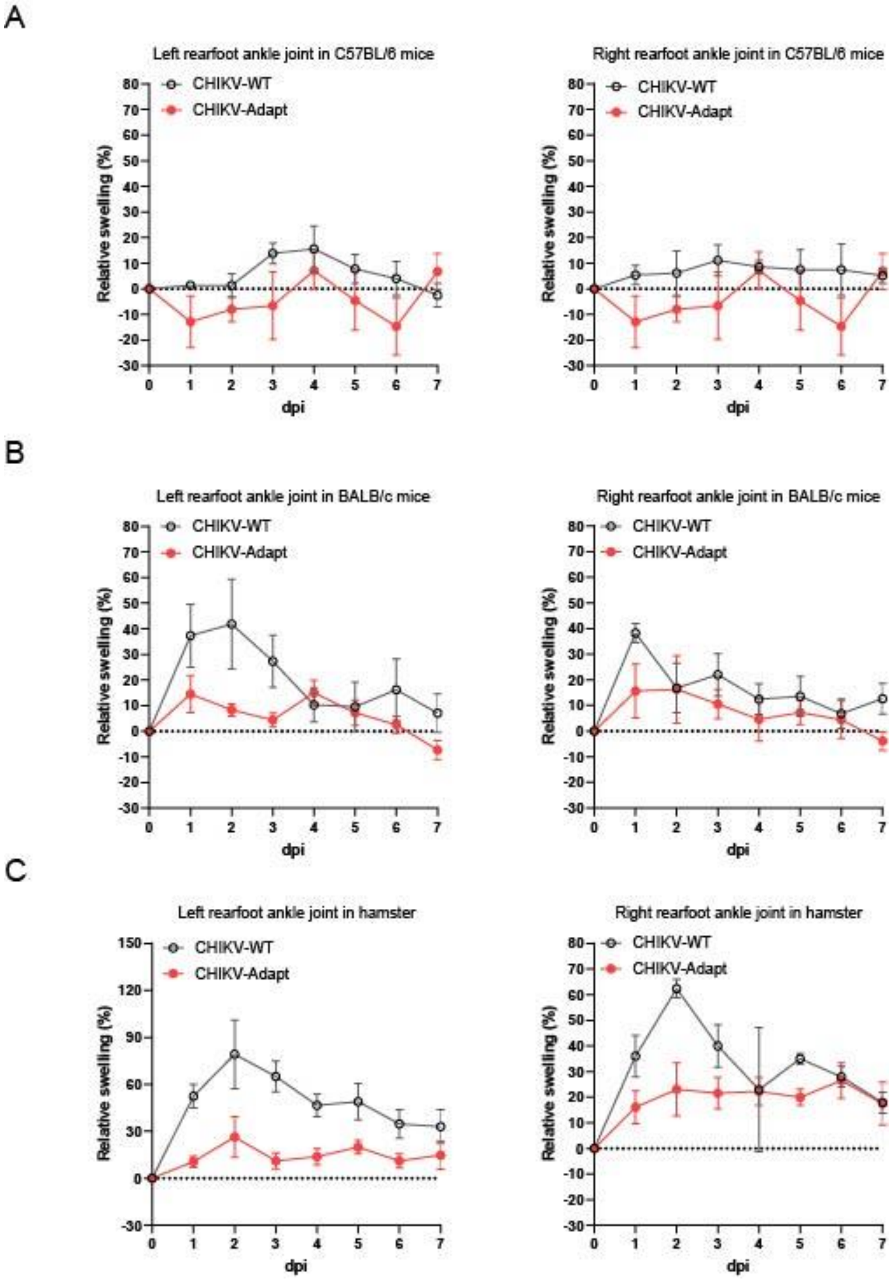


Figure S2. Rearfoot ankle joint swelling in CHIKV rodent models.

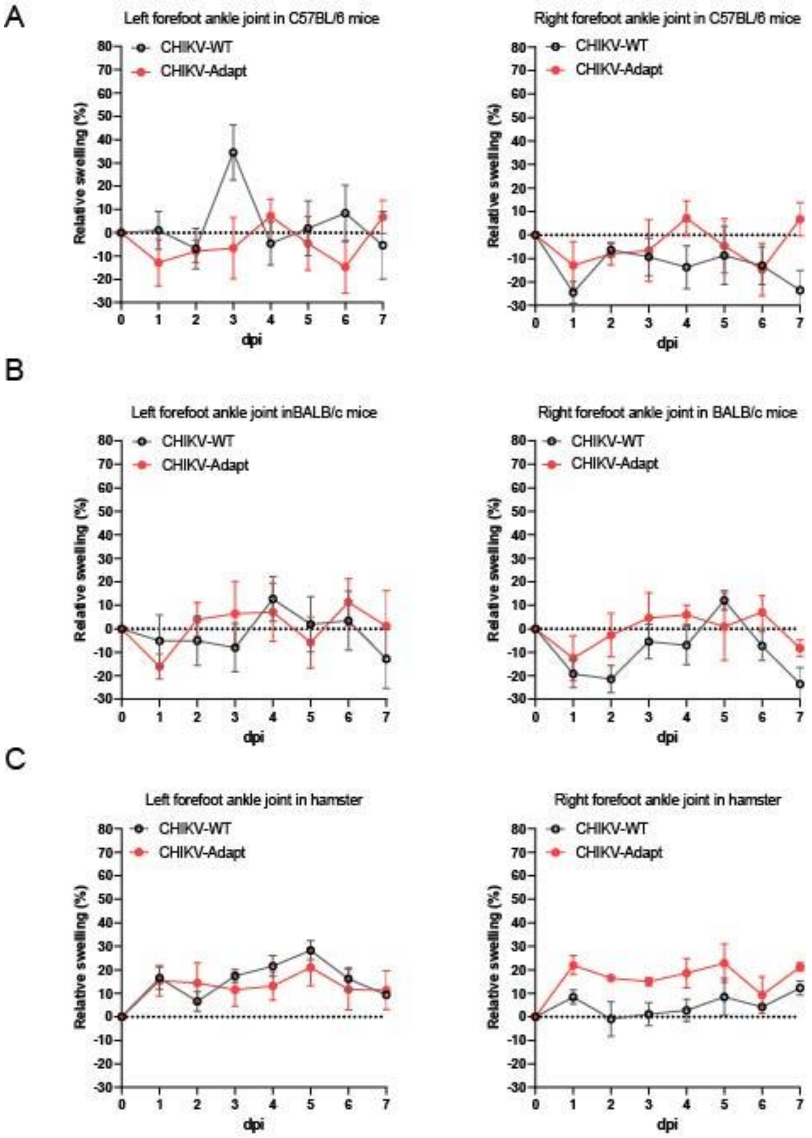


Figure S3. Forefoot ankle joint swelling in CHIKV rodent models.
